# Generalizability in two clinical trials of Lyme disease

**DOI:** 10.1186/1742-5573-3-12

**Published:** 2006-10-17

**Authors:** Daniel J Cameron

**Affiliations:** 1 Division of Medicine, First Medical Associates, 175 Main Street, Mt. Kisco, NY, 10549, USA

## Abstract

**Objective:**

To examine the generalizability of two National Institutes of Health (NIH)-funded double-blind randomized placebo-controlled clinical trials in patients with chronic Lyme disease and to determine whether selection factors resulted in the unfavorable outcomes.

**Design:**

Epidemiologic review of the generalizability of two trials conducted by Klempner et al. This paper considers whether the study group was representative of the general chronic Lyme disease population.

**Results:**

In their article in *The New England Journal of Medicine*, Klempner et al. failed to discuss the limitations of their clinical trials. This epidemiologic review argues that their results are not generalizable to the overall Lyme disease population. The treatment failure reported by the authors may be the result of enrolling patients who remained ill after an average of 4.7 years and an average of 3 previous courses of treatment. The poor outcome cited in these trials may be explained by having selected patients who had undergone delayed treatment or multiple treatments unsuccessfully. These selection factors were not addressed by the studies' authors, nor have they been discussed by reviewers. The trials have been over-interpreted by the NIH and widely publicized in a press release. The results have been extrapolated to other groups of Lyme disease patients by commentators, by a case discussant in an influential medical journal, and by health insurance companies to deny antibiotic treatment.

**Conclusion:**

The Klempner et al. trials are assumed to be internally valid based on a Randomized Control Trial (RCT) design. However, this review argues that the trials have limited generalizability beyond the select group of patients with characteristics like those in the trial. Applying the findings to target populations with characteristics that differ from those included in these trials is inappropriate and may limit options for chronic Lyme disease patients who might benefit from antibiotic treatment.

## Background

According to the Centers for Disease Control and Prevention (CDC), Lyme disease is the fastest growing vector-borne disease in the United States with over 40,000 cases reported during 2001–2002, representing a 40% annual increase in incidence [1]. Studies from the 1990's suggest that the actual number of cases may be as much as twelve times higher when factoring in underreporting [1]. Enzootic cycles of Lyme disease can be maintained in a wide range of ecologic conditions [2,3]. Forty-nine of 50 states and the District of Columbia in the USA had reported Lyme disease cases by 1998 [4] The areas of the country endemic for Lyme disease have expanded [5]. In New York, over an 11 year period, cases have spread throughout the state from the original southeastern focus [6]. Increasing numbers have also been reported in the United Kingdom, France, and Sweden [3].

Lyme disease presents formidable challenges because of the high percentage of cases that become chronic in the absence of early treatment [7,8] and the complexity and cost of managing the long-term use of antibiotics in treating chronic disease [9]. The number of Lyme disease cases that become chronic has been reported to be as low as 0.5% to 13% per year for patients treated at the time of an erythema migrans rash [10-13]. However, as many as 34% to 62% developed the chronic form of the disease in two studies [7,8]. A third of Lyme disease patients in one population-based retrospective cohort treated in the late 1980's were found to have chronic Lyme disease an average of 6.2 years after treatment [7]. Two thirds of 215 Lyme disease patients diagnosed in Westchester County, New York, USA remained ill an average of 3.2 years after treatment [8].

## Analysis

The Klempner et al. trials that appeared in the *New England Journal of Medicine *examined the benefits of treating chronic Lyme disease patients with one month of intravenous ceftriaxone followed by eight weeks of oral doxycycline [14]. Chronic Lyme disease patients who received antibiotics for 90 days were no more likely to improve than those receiving placebo. The high treatment failure rate of 60% was significantly greater than the 0–37% failure rates documented in previous studies of chronic Lyme disease [15-20] (Table 1). For this reason, it seemed prudent to examine the validity of the authors' conclusions regarding the treatment, in general, of chronic Lyme disease patients.

**Table 1:** Diversity of chronic Lyme disease studies: populations, treatments, durations and outcomes.

**Study**	**Year**	**Size**	**Patient population**	**Type of study**	**Treatment**	**Duration of Rx (days)**	**Outcome**
Dattwyler [20]	1988	23	Late Lyme disease in the NorthEast (NE) USA	Randomized trial	IV Ceftriaxone vs IV penicillin	14 vs 10	92% responded to ceftriaxone, 50% improved with penicillin
Logigian [15]	1990	27	Neurologic LD patients in NE USA	Case series	IV Ceftriaxone	14	63% improved, 22% improved but then relapsed, and 15% had no change in their condition
Hassler [19]	1990	135	Stage three borreliosis manifestations of at least six months' duration in Germany	Randomized trial	IV Cefotaxime vs IV penicillin G	10	87.9% vs 61.3% respectively Full or incomplete remission of symptoms in.
Wahlberg [17]	1994	100	Consecutive LD patients in Finland	Case series	IV Ceftriaxone, oral amoxicillin plus probenecid and/or oral cefhadroxil.*	14 to114	31% of 13 treatments successful with 14 days of IV ceftriaxone alone, 89% of 56 treatments with IV ceftriaxone followed by 100 days of amoxycillin plus probenecid successful, and 83% of 23 treatments with ceftriaxone followed by 100 days of cephadroxil successful.
Donta [18]	1997	277	Chronic LD in NE USA	Case series	Oral tetracycline	30 to 330	20% of the patients were cured; 70% of the patients' conditions improved.
Logigian [16]	1999	18	Neurologic LD in NE USA	Case series	IV ceftriaxone	28	100% of 18 patients rated themselves as back to normal or improved.
Klempner [14]	2001	107	LD with persistent symptoms in NE USA	Randomized trial	IV ceftriaxone + oral doxycycline	90	40% vs 36% for treatment vs placebo in improvement in quality of life (SF-36)
Krupp [46]	2003	55	LD with disabling fatigue in NE USA	Randomized trial	IV ceftriaxone	30	69% rx vs 23% for treatment vs placebo in the primary outcome – fatigue. No improvement on cognitive function or the clearance of Borrelia OspA antigen in the spinal fluid
Donta [25]	2003	235	LD with fatigue, musculoskeletal pain, and neurocognitive dysfunction in NE USA	Case series	Oral macrolide + hydroxychloroquine	90	80% had self reported improvements of 50% or more
Dattwyler [27]	2005	201	Late LD in NE USA	Randomized trial	IV ceftriaxone	14 vs. 28	76% vs 70% clinical cure rates for 14 and 28 days respectively
Borg [26]	2005	65	Neurologic LD in Sweden	Randomized trial	IV ceftriaxone vs oral doxycycline	10 to 14	79% vs 72% completely recovered, the remaining improved.

* Patients treated with different combinations of oral, IM or IV antibiotics and variable durations of treatment.

### Internal validity of the Lyme disease trials conducted by Klempner et al

Blinded, randomized controlled trials (RCT) are seen as the most reliable evidence in medicine if internal and external validity can be assumed [21,22]. Klempner et al. enrolled a homogeneous patient population, used a randomized design, standardized treatment, placebo-controls, blinding, a validated quality of life outcome measure, and intent-to-treat analysis. The study suggested that treatment for 3 months was no better than placebo for a select population who remained ill an average of 4.7 years after an average of more than three courses of treatment [14]. Assessing the internal validity of these trials for estimating efficacy in the study population is beyond the scope of this paper; for present purposes, results of the trials are assumed to be internally valid, based on the RCT design. However, achieving internal validity does not imply generalizability.

### Generalizability of the Lyme disease trials conducted by Klempner et al

Generalizability can be assessed by considering factors that may influence the outcome of an intervention across varied medical settings with diverse patient populations [23]. The authors of the trials did not discuss generalizability to the everyday medical setting.

Investigators of other conditions have demonstrated the consequences when a study fails to address the broader group seen in everyday clinical practice. Jüni et al. cites the lack of effectiveness of fibrinolytic therapy for suspected acute myocardial infection when generalizing the results from a younger group to the elderly and when generalizing timely fibrinolytic therapy to patients presenting more than 12 hours after symptom onset [22].

This review examines whether the Lyme disease patients enrolled in the Klempner et al. trials represent those commonly seen in everyday practice. Subjects were eligible if they were at least 18 years old, had a history of Lyme disease acquired in the United States, and had at least one of the following: a history of a single or multiple erythema skin lesion, early neurologic or cardiac symptoms attributed to Lyme disease, radiculoneuropathy, or Lyme arthritis. Documentation by a physician of previous treatment of acute Lyme disease with a recommended antibiotic regimen was also required. At the time of enrollment, all patients had one or more of the following symptoms that interfered with their function: widespread musculoskeletal pain, cognitive impairment, radicular pain, paresthesias, or dysesthesias. Profound fatigue often accompanied one of these symptoms. The chronic symptoms had to have begun within 6 months after the initial infection with B. burgdorferi and had to have persisted for at least 6 months but less than 12 years.

Patients were excluded if they had hypersensitivity to the study medication, had previously received parenteral antibiotic therapy for 60 days or more for their current symptoms, had active inflammatory synovitis, had a coexisting condition that could have accounted for their symptoms, or were unable to discontinue medication that could interfere with the evaluation of their response to the treatment regimen (e.g., narcotic analgesics or prednisone in a dose of 10 mg per day or more). Patients with a positive polymerase-chain-reaction (PCR) test for B. burgdorferi DNA in plasma or cerebrospinal fluid at baseline were also excluded.

The results of the trials are not generalizable to patients receiving treatment for chronic Lyme disease within 6 months of their initial presentation, because the investigators excluded these patients from study. Chronic Lyme disease has been reported as early as 2–4 weeks after onset of acute disease [18,24]. The impact of treatment delay is poorly understood. Asch et al. describe a retrospective cohort of 215 subjects with an average 6-week delay in getting treatment [8]. Sixty-two percent were ill an average of 3.2 years after initial treatment. The Klempner study did not consider the impact of treatment delay on long-term treatment failure. In the published report, the authors did not make it very clear that participants had already been ill with Lyme disease an average of 4.7 years at the time of their enrollment in the study [14]. Information about this potential selection factor is found only in the tables – rather than in the results section or the abstract, where it should have been in order to avoid misinterpretation of the results [14]. The Klempner report's failure to take average duration of study participants' illness into account when interpreting the results gives readers the potentially misleading impression that the Klempner et al. study can be generalized to the overall population of patients that present with persistent symptoms and a history of Lyme disease.

Furthermore, the results of the trials may not be generalizable to chronic Lyme disease patients presenting for a first or second retreatment. The authors did not discuss the significance of the study participants' average of 3 previous courses of antibiotics [14]. Again, this potential selection factor is only described in the tables, rather than, more appropriately, in the results section or the abstract [14]. Initial retreatment is both commonplace and successful in previous studies [15-20,25-27]. By failing to enroll a sufficient number of patients who had received fewer than three previous courses of treatment, the researchers may have excluded the subset of Lyme disease patients most likely to benefit from retreatment.

The investigators can only draw conclusions about the 3-month combination of oral and intravenous antibiotic treatment that was chosen for the study and not about longer treatments or simultaneous administration of multiple antibiotics. They dismissed the potential benefit of longer treatment or other combinations of antibiotics by saying "Experience with other chronic infectious diseases caused by persistent bacteria (e.g., syphilis, tuberculosis, and helicobacter infection) suggests that it is unlikely that more prolonged antibiotic therapy or a different combination of antibiotics would result in greater improvement than was observed in this study" [[14], p.89]. The authors did not provide references to support this statement; prolonged antibiotic use or simultaneous administration of multiple antibiotics *have *been effective for tuberculosis [28] and helicobacter infection [29].

The two trials cannot be generalized to chronic Lyme disease patients who have never received treatment, since these patients were excluded from study. Up to one third of patients never present with the classic erythema migrans rash, Bell's palsy, meningitis, heart block, and/or arthritis, which are indications of early Lyme disease [17,18,20,27]. It is not clear that the same treatment that is effective for early Lyme will be equally effective for untreated chronic Lyme. Prolonged antibiotic treatment may be called for in cases of untreated chronic Lyme.

The authors did not discuss the reasons two Klempner et al. trials failed whereas previous studies showed a benefit of retreatment with antibiotics [15-20]. Wells stressed the need for authors of clinical trials to keep informed about the results of other relevant studies [30]. There were six previous studies of chronic Lyme disease that described differing treatment regimens and durations, and broader populations than those included in the Klempner et al. trials (Table 1). Both oral and intravenous antibiotics were effective for late, chronic, neurologic, and stage 3 Lyme disease in Europe and the USA. The treatment duration ranged from 10 to 330 days. Given the narrowly defined study population in the Klempner et al. trials, the results of previous treatment studies should not be ignored when drawing general conclusions about effects in a broader target population.

### Misinterpretation of the results

On June 12, 2001 the National Institutes of Health (NIH) issued a press release titled "Chronic Lyme Disease Symptoms Not Helped by Intensive Antibiotic Treatment." The release quotes Klempner as follows, "We think it is unlikely that a longer course of treatment or different antibiotic combination would result in greater improvement than what we found in these studies" [[31], p.1]. The statement did not discuss the limited generalizability of the Klempner study [31].

Subsequent reviews of the trials have discouraged treatment for chronic Lyme disease without addressing their limitations. A 2002 review in *Arthritis Research *cited these trials as evidence that "Prolonged antibiotic treatment for suspected 'chronic Lyme disease syndrome' is therefore expensive, ineffective, burdened with side effects and should be avoided" [[32], p.23]. Blacklow, in a summary and comment in the *Journal Watch Infectious Diseases*, stated "it is unlikely that tinkering with antibiotic choices and durations of therapy will alter these findings" [[33], p.1]. This conclusion restates Klempner et al.'s dismissal of the value of additional antibiotic therapy or a different combination of antibiotics and even expands upon it in ways that were not suggested by the studies' results.

Several authors of other studies inappropriately cite the Klempner et al. trials to conclude that chronic Lyme disease is not infectious. Authors of a recently completed clinical trial on early Lyme disease [13] note that "There is no scientific evidence to justify prolonged antibiotic therapy for patients with any manifestation of Lyme disease, and our study and that of others [34] should further help to discourage such practice. In addition, antibiotics are no better than placebo in treating patients who carry the label of 'chronic Lyme disease,' probably because evidence indicates that this entity is not infectious" [[35], p.577]. The authors make this statement without citing evidence that supports the notion that chronic Lyme disease is not infectious, other than the Klempner trials.

In another published paper citing the Klempner et al. trials as evidence that post-Lyme syndrome is distinct from Lyme disease, patients were said to have "developed a syndrome of diffuse arthralgia, myalgia, fatigue, and subjective cognitive difficulty during or soon after LD" [[36], p.385]. However, these so-called "post-Lyme" symptoms are also typical of Lyme disease itself. The authors did not present clear evidence that "post-Lyme syndrome" was a distinct illness in patients who are demonstrably no longer infected with Lyme spirochetes.

Another author inappropriately cites the two Klempner et al. trials to support a position that Lyme disease is neither infective nor inflammatory. In an editorial commentary in the *Journal of Infectious Diseases*, Radolf considered the two trials pivotal in supporting the position that "the majority of physicians and scientists, the so-called mainstream camp, maintain that PTCLD (post-treatment chronic Lyme disease) is neither infectious nor inflammatory in nature" [[37], p.948], and that "researchers have failed to garner convincing and reproducible evidence for either persistent infection or ongoing inflammation" [[37], p.948]. Radolf cites a second Klempner et al. paper [38] derived from the same two clinical trials, stating there was no evidence of persistent or viable infection by numerous measures including cultures and PCR, CSF pleocytosis, elevated white blood count, or increased erythrocyte sedimentation rate. Neither Radolf [37] nor Klempner et al. [38] discussed the poor sensitivities of these tests for chronic Lyme disease [15]. For example, only one of a series of twenty-seven cases of neurologic Lyme disease presented with a CSF pleocytosis and that case had only 7 cells [15]. Furthermore, PCR and culture tests may only be useful for subjects with early Lyme disease who have never been treated with antibiotics [39]. Neither increased white blood count nor erythrocyte sedimentation rate is elevated in acute or chronic Lyme disease [40].

A discussant in a clinician's corner published in JAMA [41] cited the Klempner et al. trials when advising against treatment of a 58-year-old man with chronic Lyme disease. The man, who lived in the Lyme endemic area of Martha's Vineyard, had been ill for 10 years. This man had a history of Bell's Palsy and in August 1992, "he became less competent mentally. He could not do simple math and he became depressed. In 1994, he was diagnosed as having Lyme disease. At that time, he complained of neck pain radiating to his left shoulder and hand; numbness and tingling in his hand; back pain that radiated down his left leg; bilateral joint aches in both elbows and, to a lesser extent, his shoulders; bilateral tinnitus; and periodic blurred vision" [[41], p.1002]. The man was treated with prednisone in 1992. Antibiotic treatment was delayed until 1994. The symptoms improved with repeated courses of oral tetracycline and clarithromycin, only to recur. The discussant cited the Klempner et al. trials as evidence against the value of further antibiotic treatment. Instead, he advised treatment for fibromyalgia even though he admitted that the patient did not meet the criteria for this condition. Fibromyalgia treatment has been disappointing for people with this kind of history [42].

Finally, two health insurance companies cite the Klempner et al. trials as justification for not covering treatment with intravenous antibiotics. One company policy states that they "will not cover IV therapy beyond 28 days for Lyme Disease without review and input from a trained Infectious Disease Specialist approved by GHI-HMO." Furthermore, the company "will not cover IV therapy for Lyme Disease for Chronic Lyme Disease or Post-Lyme Disease Syndrome without input from a trained Infectious Disease Specialist approved by GHI-HMO " [43]. Citing the Klempner et al. trials, [44] Cigna does not cover any treatment for patients with persistent symptoms and a history of Lyme disease, unless recurrent arthritis, central nervous system (CNS), or peripheral nervous system involvement can be demonstrated. Treatment for chronic Lyme disease would otherwise be considered experimental, investigational, or unproven and therefore not covered, resulting in limited treatment options for many patients who might have benefited from additional antibiotics.

## Conclusion

Klempner et al. did not adequately critique the generalizability of their trials. This review argues that the study participants were not representative of the overall population of chronic Lyme disease patients that present with persistent symptoms and a history of disease. Limited generalizability has been a problem of other randomized trials [21] and it remains one here.

This review argues that the poor treatment response in the Klempner et al. trials may be explained by having selected patients who had undergone delayed treatment or multiple treatments unsuccessfully. The quality of life of subjects enrolling in the Klempner et al. trials was worse than that of the average type II diabetic or patient recovering from a heart attack, and as poor as that of subjects suffering from congestive heart failure [14]. In other words, it may be an example of offering patients "too little too late."

Klempner himself described his concerns about the study population to the editors of *Science: *"After a year of advertising, only 57 subjects had been enrolled. The goal is to get 260 by the time the study ends in 2 years. More than 1200 people have expressed interest, and 700 have come in for screening. But only 1 in 10 who appear in the clinic fits the study's strict criteria" [45, p.1431]. Neither in the *Science *interview [45], nor when reporting the trials' results in the New England Journal of Medicine [14], did Klempner discuss whether the strict criteria was a factor leading to the average 4.7 year onset of illness of subjects enrolled.

Two additional randomized trials and one case series have been published since the 2001 Klempner et al. trials [25-27]. A fourth trial by Krupp supported antibiotic treatment for a subset of chronic Lyme disease patients with fatigue [46]. One month of intravenous ceftriaxone was effective at reducing the primary fatigue endpoint but not the secondary endpoints of cognitive function or OspA antigen [46]. These additional trials of chronic Lyme disease [25, 26, 27, 46] continue to suggest that treatment may be beneficial for some subgroups of patients who were not well represented in the Klempner et al. trials.

In summary, this review exposes the limited generalizability of the findings of Klempner et al., and the overreaching impact these trials have had on influencing policies that affect unrepresented patient groups. In interpreting the results of these trials, physicians should consider the select group of patients that were chosen for study and whether the patients in their care might respond differently to treatment.

## Competing interests

The author is a clinician who treats Lyme diseaseand an advocate ofproviding moreaccurate information about treatment options. He has no interests that conflict with that goal.
